# Factors associated with late initiation of antiretroviral therapy in Iran’s HIV/AIDS surveillance data

**DOI:** 10.1038/s41598-023-50713-0

**Published:** 2024-01-02

**Authors:** Mehdi sharafi, Alireza Mirahmadizadeh, Jafar Hassanzadeh, Mozhgan Seif, Alireza Heiran

**Affiliations:** 1 Social Determinants in Health Promotion Research Center, Hormozgan Health Institute, Hormozgan University of Medical Sciences, Bandar Abbas, Iran; 2 Non-communicable Diseases Research Center, Shiraz University of Medical Sciences, Shiraz, Iran; 3 Department of Epidemiology, Research Centre for Health Sciences, Institute of Health, School of Health, Shiraz University of Medical Sciences, Shiraz, Iran; 4 Non-communicable Diseases Research Center, Department of Epidemiology, School of Health, Shiraz University of Medical Sciences, Shiraz, Iran

**Keywords:** Diseases, Health care, Medical research, Risk factors

## Abstract

Early initiation of Antiretroviral Treatment (ART) in HIV patients is essential for effectively suppressing the viral load and prognosis. This study utilized National HIV/AIDS Surveillance Data in Iran to identify factors associated factors with the duration to initiate ART. This hybrid cross-sectional historical cohort study was conducted on Iran’s National HIV/AIDS Surveillance Data from 2001 to 2019. Sociodemographic characteristics, route of transmission, HIV diagnosis date, and ART initiation date were collected. Multivariable linear and quantile regression models were employed to analyze the duration to initiate ART by considering predictor variables. This study included 17,062 patients (mean age 34.14 ± 10.77 years, 69.49% males). Multivariate quantile regression coefficients varied across different distributions of the dependent variable (i.e., duration to initiate ART) for several independent variables. Generally, male gender, injecting drug use (IDU), and having an HIV-positive spouse were significantly associated with an increased duration to initiate ART (p < 0.05). However, a significant decrease was observed in older patients, those with a university level education, men who had sex with men (MSM), and patients diagnosed after 2016 (p < 0.05). Despite improvements in the duration to initiate ART after implementing the WHO’s 2016 program in Iran, various sociodemographic groups were still vulnerable to delayed ART initiation in the region. Therefore, programs including early testing, early ART initiation, active care, educational and cultural interventions, and appropriate incentives are required for these groups.

## Introduction

The HIV pandemic is a significant global public health challenge^1^. Despite a decrease in new HIV cases worldwide, the number is increasing in Iran^2^. UNAIDS estimates indicate 59,000 People Living With HIV (PLWH) in Iran, with 4100 new cases and 2500 AIDS-related deaths annually^2^. Additionally, despite attempts, approximately two-thirds and one-third of HIV patients in Iran are diagnosed late and in advanced disease, respectively^3^. Although injecting drug use (IDU) is still the most common transmission route, Iran's HIV epidemic is shifting toward sexual contact^2^.

Antiretroviral Treatment (ART) since 1996 has substantially improved outcomes for PLWH, including suppressing viral loads to an undetectable level, restoring CD4+ T-cell counts, and decreasing AIDS-related morbidity and mortality^4–7^. Additionally, early ART initiation is associated with a higher probability of good prognosis^8^, serving as the main predictor of virological suppression^9^, and can substantially decline HIV transmission rate by 96% in couples with one HIV-infected partner, compared to late ART initiation^10,11^. Conversely, late or delayed ART initiation in linked to weakened immune function, clinical progression, increased risk of opportunistic infections, and higher AIDS and non-AIDS related morbidity and mortality among PLWH, as well as higher pressure on healthcare systems^12–15^.

One of the goals of 90.90.90 UNADIS is to suppress viral loads in 90% of treated patients. To achieve this goal, early initiation of ART has a crucial role^16^. The "treat-all" strategy, initiating ART regardless of CD4+ count, was implemented globally in 2015 to improve HIV treatment outcomes^17^, boosting global ART coverage in the following year (i.e., 48% in 2015, 53% in 2016, and 59% in 2017)^18^. Nevertheless, a substantial proportion of patients still initiate ART at an advanced HIV disease^19–21^, and, despite these high-cost efforts, the underlying causes are not well understood^22–24^.

Various risk factors are reported for late ART initiation, including socioeconomic inequalities, disease-related stigma, male sex, older age, low education, unemployment, co-morbidities, IDU, early initiation in the calendar year, medication side effects, and psychological issues^19,25–31^. In addition, despite improved ART availability and affordability, the ‘‘treat-all’’ scheme is not mandatory in many regions, leading newly diagnosed PLWH to opt against ART due to feeling healthy or fearing stigma and discrimination^23^.

Iran is one of the active countries battling HIV/AIDS in the Middle East, initiated HIV surveillance since 2001^32^. The HIV/AIDS case-based surveillance is the central part of the National HIV/AIDS surveillance system initiated in 2009^2^. Nonetheless, information is limited among Iranian late-initiating HIV patients. This study aimed to explore several potential factors influencing the duration between HIV diagnosis and ART initiation—defined as late ART initiation—using the Iran’s National HIV/AIDS Surveillance Data during 2001–2019.

## Materials and methods

### Study design, population and data acquisition

This hybrid cross-sectional historical cohort study included all individuals registered in the Iran’s National HIV/AIDS Surveillance Database from 2001 to 2019 (N = 18,100). Behavioral disease counseling centers affiliated with medical universities and non-profit centers are mandated to report new cases to this database for routine HIV care according to the national protocol. The registry mission is to act as the central hub for recording the registered patients’ demographic characteristics and high-risk behaviors, medical counseling services, HIV screening and testing, support services, and routine HIV cares and follow-up, including ART, periodic physician visits, CD4 cell count measurement, viral load testing, and evaluation of tuberculosis, hepatitis C virus (HCV), hepatitis B virus (HBV), and other comorbidities. These measures are recorded in the embedded data management software in the Ministry of Health. The anonymous data then will be sent to the counseling centers for behavioral disorders and the Iranian Center for Infectious Disease Control and Prevention (CDC)^33^. The research team obtained anonymized data in spreadsheet format upon request to the registry center in the Ministry of Health after receiving ethical approval from the local Research Ethics Committee of Shiraz University of Medical Sciences (IR.SUMS.SCHEANUT.REC.1400.045).

### Inclusion and exclusion criteria

The study included individuals receiving ART without age or sex restrictions (N = 18,100). Exclusion criteria comprised missing values for diagnosis date (N = 310) or ART initiation date (N = 321), as late ART initiation could not be computed, and any missing values for selected independent variables (N_transmission route_ = 199, N_age_ = 98, N_education level_ = 110).

### Included variables

The dependent variable was the duration to initiate ART, considering the WHO’s 2017 recommendation for ART initiation on the same day as HIV diagnosis, known as the "test and treat" policy. Every additional day needed to initiate ART after the diagnosis day was considered a failure, indicating 1 day of late ART initiation^34^. Accordingly, the variable was not categorized. Independent variables included demographic factors (age, sex, education level), year of diagnosis, and transmission route (e.g., IDU, heterosexuality, having an HIV/AIDS-positive spouse, having a spouse with high-risk behaviors, men who have sex with men (MSM), and other transmission routes).

### Statistical analysis

The mean, a frequently used measure of central tendency, may not always be the most suitable indicator due to its susceptibility to outliers and non-normal distribution. Conversely, the median, being robust and unaffected by outliers and distribution type, yields a more reliable alternative. Standard regression, such as linear regression, which minimizes the distance from the mean, shares these limitations. The least absolute deviation technique, minimizing the distance to the median, addresses these issues and is considered robust. Extending this technique to other quintiles results in quintile regression, which provids robust estimates for different quantities and offers valuable insights into the data relationship^35,36^.

In this study, quantile regression was used to assess the association between various independent variables and the dependent variable, the duration to initiate ART. This choice was motivated by two reasons: the non-normal distribution of the dependent variable (Supplementary file: Fig. 1) and the assumption that various independent variables might exhibit varying degrees of association with different quantiles of the dependent variable, which cannot be fully captured by the single coefficient given by linear regression models.

Statistical analyses were carried out using Stata software, version 15. Patient characteristics were described in terms of frequency and percentage for qualitative variables, and as mean and standard deviation (SD) or median and interquartile range (IQR) for quantitative variables. Model outputs were reported through β coefficients with 95% confidence intervals (CIs), and the significance level was set at α = 0.05 for all tests.

Initially, univariable and multivariable linear regression models were performed to assess the associations between the duration to initiate ART and independent variables. Independent variables with P statistics less than 0.2 in univariable analyses were selected to enter the multivariable linear model (Supplementary file: Table 1). Subsequently, multivariable quantile regression was used to investigate the obtained associations in more detail. For each independent variable, regression coefficients were reported separately at the 25th, 50th, and 75th percentiles of the duration to initiate ART (i.e., q25, q50, q75). Additionally, the Powell kernel estimator was used to ensure normality and consistency for the asymptotic variance–covariance matrix of the quantile regression^37^.

### Ethical approval and consent to participate

Ethical issues including plagiarism, informed consent, misconduct, data fabrication and/or falsification, double publication and/or submission, redundancy, etc. were completely observed by the authors. This study was performed according to the ethical guidelines expressed in the Declaration of Helsinki and the Strengthening of the Reporting of Observational Studies in Epidemiology (STORB) guideline. The study was also approved by the Research Ethics Committee of Shiraz University of Medical Sciences (IR.SUMS.SCHEANUT.REC.1400.045). Informed consent was also waived by the Research Ethics Committee of Shiraz University of Medical Sciences (IR.SUMS.SCHEANUT.REC.1400.045).

## Results

### Basic characteristics of the study population

After excluding 1038 patients based on various exclusion criteria, the study involved 17,062 patients, with an average age of 34.14 ± 10.77 years, including 11,856 males (69.49%). The majority of patients had an education level up to junior high school (N = 4954; 29.04%) and elementary school (N = 4217; 24.72%). In addition, 12,357 patients (72.42%) had been diagnosed before 2016. About high-risk behaviors, 7308 (42.83%) and 5772 (33.83%) individuals were identified as IDUs and heterosexual, respectively. The median duration to initiate ART was 550 days (IQR: 62, 1793.5) for patients diagnosed before 2016 and 25 days (IQR: 9, 61) for those diagnosed after 2016, showing a statistically significant difference (p < 0.001). Detailed characteristics of the study population are presented in Table 1.

**Table 1:** Characteristics of the study population.

Variables	Group	Frequency (%)
Gender	Male	11,856 (69.49)
	Female	5206 (30.51)
Education level	Illiterate	2914 (17.08)
	Elementary	4217 (24.72)
	Junior high school	4954 (29.04)
	Senior high school	3615 (21.19)
	Higher-than-diploma	1362 (7.98)
Year of report	Before 2016	12,357 (72.42)
	After 2016	4705 (27.58)
Injecting drug user	No	9754 (57.17)
	Yes	7308 (42.83)
Men who have sex with men (MSM)	No	16,231 (95.13)
	Yes	831 (4.87)
Heterosexual	No	11,290 (66.17)
	Yes	5772 (33.83)
Have an HIV-positive spouse	No	14,252 (83.53)
	Yes	2810 (16.47)
Having a spouse with high-risk behaviors	No	16,239 (95.18)
	Yes	823 (4.82)
Other routes of transmission^a^	No	13,983 (81.95)
	Yes	3079 (18.05)
Age at diagnosis, *year*^b^	34.14 ± 10.77	
Duration to initiate ART, *day*^b^	824.85 ± 1220.54	

^a^Blood transfusion, vertical transmission, work-related.

^b^Mean ± SD.

### Results of multivariable linear regression model

Initially, a linear regression model was employed to explore factors associated with the duration to initiate ART. The results are shown in Table 2. The mean duration to initiate ART was significantly shorter in females than in males (β = − 226.75 days [95% CI − 273.99, − 1789.52], p < 0.001). Higher education levels were associated with a reduction in the mean duration to initiate ART, with statistically significant decreases observed for patients with elementary and junior high school degrees compared to illiterate individuals (β_junior high school_ = 148.68 days [95% CI 98.21, 199.15], p < 0.001; β_elementary_ = 179.41 days [95% CI 127.6, 231.13], p < 0.001). Patients diagnosed after 2016 experienced a significant reduction in the mean duration to initiate ART by 855.091 days compared to those diagnosed before 2016 (95% CI − 893.63, − 816.55, p < 0.001). Additionally, an increase in age significantly decreased the mean duration to initiate ART by 18.28 days (95% CI − 19.85, − 16.70, p < 0.001). IDUs had a significantly increased duration to initiate ART by 326.73 days (95% CI 285.31, 368.15, p < 0.001). Moreover, the mean duration to initiate ART decreased by 123.01 days in MSM group compared to the non-MSM group (95% CI − 201.15, − 44.88, p = 0.002). In the patients who had an HIV-positive spouse, the mean duration to initiate ART increased by 125.95 days (95% CI 65.66, 186.25, p < 0.001). Finally, the mean duration to initiate ART reduced by 159.47 days in patients with other transmission routes (95% CI − 205.01, − 113.93, p < 0.001). However, this association was not significant among heterosexuals and patients with high-risk spouses (p > 0.05).

**Table 2:** Adjusted linear regression for determining the association between the independent variables and duration (days) to initiate ART.

Variables	Group	Adjusted coefficient	SE	95% CI	P
Gender	Male	Reference	–	–	–
	Female	− 226.75	24.09	− 273.99, − 1789.52	< 0.001
Education level	Illiterate	Reference	–	–	–
	Elementary	179.41	26.38	127.6, 231.13	< 0.001
	Junior high school	148.68	25.74	98.21, 199.15	< 0.001
	Senior high school	30.07	27.48	− 23.80, 83.94	0.274
	Higher-than-diploma	− 57.97	36.62	− 129.76, 13.81	0.113
Years of report	Before 2016	Reference	–	–	–
	After 2016	− 855.091	19.66	− 893.63, − 816.55	< 0.001
Age	–	− 18.28	0.80	− 19.85, − 16.70	< 0.001
Injection drug user	No	Reference	–	–	–
	Yes	326.73	21.13	285.31, 368.15	< 0.001
Men who have sex with men (MSM)	No	Reference	–	–	–
	Yes	− 123.01	39.86	− 201.15, − 44.88	0.002
Heterosexual	No	Reference	–	–	–
	Yes	− 32.68	19.14	− 70.20, 4.84	0.088
Having an HIV-positive spouse	No	Reference	–	–	–
	Yes	125.95	30.76	65.66, 186.25	< 0.001
Having a spouse with high-risk behaviors	No	Reference	–	–	–
	Yes	− 45.39	43.54	− 130.74, 39.96	0.297
Other routes of transmission^a^	No	Reference	–	–	–
	Yes	− 159.47	23.23	− 205.01, − 113.93	< 0.001

^a^Blood transfusion, vertical transmission, work-related.

### Results of multivariable quantile regression model

**Figure 1:**
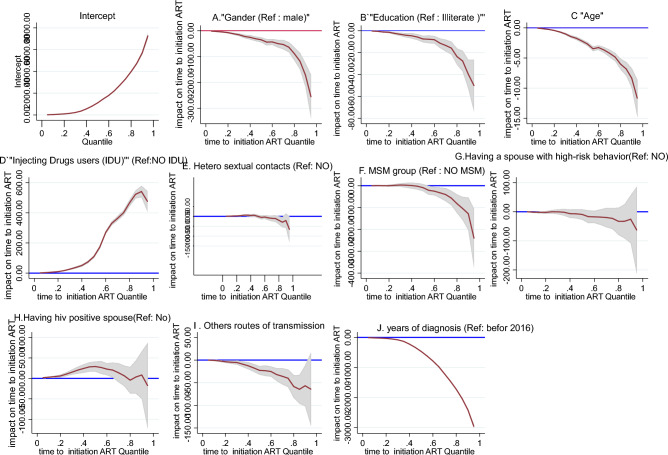
(**A**–**J**) Multivariable quantile regression between duration to initiate ART and various independent variables amongst HIV patients in Iran [Note: The x-axis represents the quantiles of the distribution of duration to initiate ART and the y-axis represents the change in duration to initiate ART associated with a one-unit change in the studied independent variable, with holding other covariates constant. This change is considered significant at a particular quantile when the associated 95% CI (shaded area) does not cross the 0 value (blue line)].

In addition to the multivariable regression model, a multivariable quantile regression model was employed to further investigate the above-mentioned relationships in various quantiles (q25, q50 and q75) of the duration to initiate ART. Additionally, each pair of associations was visualized through the graph of quantile coefficients (Fig. 1A–J).

The results showed a statistically significant shorter duration to initiate ART among females compared to men in all quantiles, representing a constantly significant shorter duration to initiate ART among females (q25: − 11.93 days [95% CI − 15.64, − 8.23], p < 0.001; q50: − 42.32 days [95% CI − 56.79, − 27.86], p < 0.001; q75: − 75.5 days [95% CI − 101.43, − 49.66], p < 0.001) (Table 3, Fig. 1A).

**Table 3:** Predictors of duration (days) to initiate ART using multivariate quantile regression analysis.

Variables	Group	First quintile, q (25)			Second quartile, q (50)			Third quartile, q (75)		
		Coefficient [95% CI]	SE^b^	P	Coefficient [95% CI]	SE^b^	P	Coefficient [95% CI]	SE^b^	P
Gender	Male	Reference	–	–	–	–	–	–	–	–
	Female	− 11.93 [− 15.64, − 8.23]	1.89	< 0.001	− 42.32 [− 56.79, − 27.86]	7.38	< 0.001	− 75.5 [− 101.43, − 49.66]	13.18	< 0.001
Education level	Illiterate	Reference	–	–	–	–	–	–	–	–
	Elementary	9.2 [4.72, 13.67]	2.28	< 0.001	63.60 [37.50, 89.71]	13.32	< 0.001	81 [44.27, 117.73]	18.74	< 0.001
	Junior high school	5.6 [1.27, 9.93]	2.21	0.011	57.61 [29.13, 86.10]	14.53	< 0.001	62 [19.96, 104.04]	21.45	0.004
	Senior high school	− 0.8 [− 3.70, 2.10]	1.48	0.588	22.48 [0.97, 43.98]	10.97	0.040	− 4.5 [− 31.14, 22.14]	13.59	0.741
	Higher-than-diploma	− 6 [− 10.62, − 1.38]	2.36	0.011	3.18 [− 25.37, 31.73]	14.56	0.827	− 27 [− 60.89, 6.89]	17.29	0.118
Year of report	Before 2016	Reference	–	–	–	–	–	–	–	–
	After 2016	− 50.27 [− 54.59, − 44.94]	2.72	< 0.001	− 463.73 [− 494.67, − 432.81]	15.78	< 0.001	− 1438 [− 1499.44, − 1376.56]	31.34	< 0.001
Age	–	− 0.733 [− 0.87, − 0.60]	0.07	< 0.001	− 3.44 [− 4.28, − 2.59]	0.43	< 0.001	− 6 [− 7.78, − 4.21]	0.91	< 0.001
Injection drug use	No	Reference	–	–	–	–	–	–	–	–
	Yes	16.93 [13.33, 20.54]	1.84	< 0.001	113.95 [77.86, 150.05]	18.41	< 0.001	386.5 [308.53, 464.47]	39.78	< 0.001
Men who have sex with men (MSM)	No	Reference	–	–	–	–	–	–	–	–
	Yes	2.33 [− 2.29, 6.96]	2.36	0.323	− 5.64 [− 19.59, 8.30]	7.11	0.428	− 58.5 [− 81.86, − 35.14]	11.92	< 0.001
Heterosexual	No	Reference	–	–	–	–	–	–	–	–
	Yes	2.73 [0.23, 5.24]	1.28	0.033	− 2.08 [− 13.42, 9.26]	5.79	0.719	− 18 [− 40.98, 4.98]	11.73	0.125
Having an HIV-positive spouse	No	Reference	–	–	–	–	–	–	–	–
	Yes	10.73 [6.05, 15.41]	2.39	< 0.001	34.38 [18.70, 50.05]	8.00	< 0.001	8.5 [− 10.47, 27.47]	9.68	0.380
Having a spouse with high-risk behaviors	No	Reference	–	–	–	–	–	–	–	–
	Yes	− 1 [− 8.90, 6.90]	4.03	0.804	− 13.97 [− 40.93, 13.00]	13.76	0.310	− 32.5 [− 57.16, − 7.84]	12.58	0.010
Other routes of transmission^b^	No	Reference	–	–	–	–	–	–	–	–
	Yes	− 4.6 [− 8.11, − 1.09]	1.79	0.010	− 24.76 [− 38.42, − 11.10]	6.97	< 0.001	− 55.5 [− 88.32, − 22.68]	16.74	0.001

^a^SE, Bootstrap standard error.

^b^Blood transfusion, vertical transmission, work-related.

In terms of education level, with illiterate education status as the reference group, the association was more prominent in the groups with elementary and junior high school education levels; that is, there was a constantly significant longer duration to initiate ART among those with elementary education level (q25: 9.2 days [95% CI 4.72, 13.67], p < 0.001; q50: 63.60 days [95% CI 37.50, 89.71], p < 0.001; q75: 81 days [95% CI 44.27, 117.73], p < 0.001) and those with a junior high school education level (q25: 5.6 days [95% CI 1.27, 9.93], p = 0.011; q50: 57.61 days [95% CI 29.13, 86.10], p < 0.001; q75: 62 days [95% CI 19.96, 104.04], p = 0.004), compared to illiterate patients. No significant difference was observed between patients with senior high school or higher-than-diploma education level and the reference group in terms of duration to initiate ART, except for a significantly longer duration at q50 in senior high school group (22.48 days [95% CI 0.97, 43.98], p = 0.040) and a significantly shorter duration at q25 in higher-than-diploma group (− 6 days [95% CI − 10.62, − 1.38], p = 0.011). It is noteworthy that the higher-than-diploma group had the shortest duration to initiate ART at q25 and q75 of dependent variable, compared to the reference group (Table 3, Fig. 1B).

The duration to initiate ART in patients diagnosed after 2016 was constantly shorter than those diagnosed before 2016, according to significant effect sizes in different quantiles (q25: − 50.27 days [95% CI − 54.59, − 44.94], p < 0.001; q50: − 463.73 days [95% CI − 494.67, − 432.81], p < 0.001; q75: − 1438 days [95% CI − 1499.44, − 1376.56], p < 0.001) (Table 3, Fig. 1J). An important point is that this variable had the largest effect sizes for shorter durations to initiate ART among the studied independent variables.

Every 1-year increase in age was significantly associated with a shorter duration to initiate ART, a consistent finding across all quantiles (q25: − 0.733 days [95% CI − 0.87, − 0.60], p < 0.001; q50: − 3.44 days [95% CI − 4.28, − 2.59], p < 0.001; q75: − 6 days [95% CI − 7.78, − 4.21], p < 0.001) (Table 3, Fig. 1C).

Different routes of transmission had varying effects, in terms of the intensity and direction of association, on the duration to initiate ART. IDUs constantly had a significantly longer duration to initiate ART compared to non-IDUs (q25: 16.93 days [95% CI 13.33, 20.54], p < 0.001; q50: 113.95 days [95% CI 77.86, 150.05], p < 0.001; q75: 386.5 days [95% CI 308.53, 464.47], p < 0.001) (Table 3, Fig. 1D). Remarkably, IDUs had the largest effect sizes affecting the durations to initiate ART between different routes of transmission. Interestingly, MSMs showed no significant difference in the duration to initiate ART compared to non-MSMs at q25 and q50; however, they had a significantly lower probabilities for high durations to initiate ART (q75) compared to non-MSMs (− 58.5 days [95% CI − 81.86, − 35.14], p < 0.001) (Table 3, Fig. 1F). In addition, being heterosexual posed a subtle association with the duration to initiate ART (Table 3, Fig. 1E). Moreover, individuals with an HIV/AIDS positive spouse constantly had a longer duration to initiate ART compared to those who had not, in different quantiles; although, this association was only significant at q25 (10.73 days [95% CI 6.05, 15.41], p < 0.001) and q50 (34.38 days [95% CI 18.70, 50.05], p < 0.001) (Table 3, Fig. 1H). Furthermore, the groups of having a high-risk spouse and other routes of transmission constantly showed a shorter duration to initiate ART, compared to their counterparts; however, these effects were only statistically significant at q75 for the first group (− 32.5 days [95% CI − 57.16, − 7.84], p = 0.010) (Table 3, Fig. 1G) and at all quantiles for the second group (q25: − 4.6 days [95% CI − 8.11, − 1.09], p = 0.010; q50: − 24.76 days [95% CI − 38.42, − 11.10], p < 0.001; q75: − 55.5 days [95% CI − 88.32, − 22.68], p = 0.001) (Table 3, Fig. 1I).

## Discussion

The present study was performed on the Iran’s National HIV/AIDS Surveillance Data, employing quantile regression analysis, to identify the factors influencing the duration to initiate ART. The key findings are outlined as follows: firstly, the duration to initiate ART for patients who diagnosed after 2016 constantly remained shorter than those diagnosed before 2016, with the largest effect sizes observed for shorter durations to initiate ART among the studied independent variables. Secondly, females had a statistically significant shorter duration to initiate ART compared to men across all quantiles. Thirdly, older ages were significantly associated with shorter duration to initiate ART, in all quantiles. Fourthly, individuals with a higher-than-diploma education level had the shortest duration to initiate ART at q25 and q75 of dependent variable, compared to illiterate patients. Intriguingly, those with elementary or junior high school education levels constantly experienced a significantly higher duration to initiate ART compared to illiterate patients. Lastly, various transmission routes had diverse effects on the duration to initiate ART. IDUs constantly had a significantly larger duration to initiate ART compared to non-IDUs, with the largest effect sizes among different transmission routes. Additionally, MSMs did not show a significant difference in duration to initiate ART compared to non-MSMs at q25 and q50; however, they had a significantly shorter duration at q75. Moreover, individuals with an HIV/AIDS positive spouse had a larger duration at q25 and q50. Furthermore, groups with high-risk spouses and other transmission routes constantly showed a significantly shorter duration at q75 and all quantiles for the first and second groups, respectively. Heterosexual transmission showed a subtle association with the duration to initiate ART.

We found that patients who diagnosed after 2016 had a shorter duration to initiate ART compared to those diagnosed before 2016. In accordance with this finding, a study from southern Iran on 1,326 adult HIV patients showed that initiating ART during 2011–2013 had 3.65 odds [95% CI 2.28, 5.86] for late ART initiation compared to more recent years (2018–2021)^38^. Additionally, Lahuerta et al.^28^ and Ndawinz et al.^39^ found that patients initiating ART in recent calendar years had lower odds of late ART initiation Moreover, a study from China showed a constant decline in delayed ART initiation from 2010 to 2020, with a sharp decrease in 2016 (58.0% to 36.9%), coinciding with national guideline reforms^40^. In contrast, several studies reported no change or an increasing trend in late-initiating patients, reflecting challenges in implementing the ‘‘treat-all’’ scheme. Lee et al.^41^ noted a significant decrease in late ART initiation from 77.14% in 2009 to 39.1% in 2019, with a slight increase from 2015 to 2019 (33.03% to 37.80%). In Brazil, Pacheco et al.^12^ observed an increase from 2014 to 2018 despite a decreasing trend in previous years. Monitoring how individuals initiate treatment reflects the success of HIV care in a region. Our findings suggest the significant progress of Iran’s health system in improving HIV diagnosis and treatment, particularly after implementing the 2016 WHO program. Iran’s HIV/AIDS prevention and control program, spanning four main programs, has evolved to focus on achieving the UNAIDS 90-90-90 targets^2^. A comparison of mean duration to initiate ART before and after these goals shows a significant decrease.

In quantile regression, females constantly had a statistically significant shorter duration to initiate ART compared to men across all quantiles, aligning with previous studies. Lahuerta et al.^28^ reported that individuals older than 45 years had lower odds of late ART initiation in Mozambique (aOR: 0.72 [95% CI 0.67, 0.77]). A study on 1180 Ethiopian adults found that being male was associated with higher odds of late ART initiation (aOR: 2.02 [95% CI 1.50, 2.73])^42^. Another study in Iran also observed similar findings (OR: 0.41 [95% CI 0.31, 0.55])^38^. The lower likelihood of late ART initiation in females could be justified by routine testing and care during antenatal visits provided by Prevention of Mother to Child Transmissions (PMTCT) services^43–45^, and differences in health-seeking behavior^46^.

Considering age, an increase resulted in a significant decrease in the mean duration to initiate ART across all quantiles. This contradicts most previous studies reporting older age as a risk factor for late ART initiation^19,41,43,47–49^, attributed to easier access to care for younger individuals^43^ and a lower perceived risk for HIV in elderly individuals^50^. For example, a study in Taiwan showed significantly higher odds for late initiating ART in the 31–40 years and > 40 years age groups compared to the < 30 years age group (adjusted OR (aOR): 1.53 [95% CI 1.12, 2.08], 2.49 [95% CI 1.53, 4.03], respectively). Another study from Spain showed that for every 10 years increase in age, there was a 10% (aHR: 1.10 [95% CI 1.10, 1.20]) increase in ART initiation^51^. However, a study in China found that older age was significantly associated with a decreased risk of delayed ART initiation, defined as initiating ART more than 1 month after diagnosis; although, the risk was increased in respect to late ART initiation (CD4 cell count < 200 cells/μL or clinical AIDS diagnosis). In addition, a study in Cameroon reported late ART initiation among young males eligible for ART, but attributed it to delays in duration from ART eligibility to ART initiation rather than later presentation for HIV care^52^.

In our study, highly educated individuals (i.e., higher-than-diploma) had a lower likelihood of late ART initiation compared to illiterate patients, aligning with previous studies^26,50^. The COHERE study, covering nine HIV cohorts from six European countries, showed strong associations between low education levels and delayed HIV diagnosis and late ART initiation^53^. Rodrigues et al.^26^ found that HIV adult patients with lower educational levels had higher odds for late ART initiation compared to those with university degrees. This finding has been also reported in low-income regions. For example, a study of 36,411 adult patients who initiated ART during 2005–2009 in Mozambique showed a significantly lower likelihood of late ART initiation among patients with secondary of higher educational levels compared to those with primary or lower levels (aOR: 0.87 [95% CI 0.83, 0.93])^28^. Also, a study on 417 PLWHs from Western Ethiopia showed that lower educational levels were significantly associated with late ART initiation. The timely initiation of ART can be affected by the health-seeking behavior and the level of knowledge about the benefits of early ART initiation, which in turn might be partially explained by the education level of the individuals, a proxy of socio-economic status^29^. Hence, although ART is freely available in Iran, patients’ low awareness and literacy might prevent attendance and adherence to treatment^54^. Moreover, interestingly, we observed that patients with elementary or junior high school education levels constantly had a significantly longer duration to initiate ART, compared to the illiterate patients. This finding has been also reported by a study of 412 HIV patients in Northwest Ethiopia. Anlay et al.^48^, showed adult patients with secondary and tertiary school education levels had 2.59 and 3.28 odds for late ART initiation, compared to the illiterate patients, respectively. Nonetheless, such varying associations between different education levels and duration to initiate ART might be multifactorial.

IDUs and HIV patients who had HIV-positive spouses had a significantly increased duration to initiate ART compared to their counterparts, with IDUs having the highest effect sizes among different transmission routes. In Iran, the majority of HIV patients are male IDUs^55^. Based on a study conducted on this group in Iran, Ghalekhani et al.^56^, showed that 57% of IDUs had received care services and initiated ART, with only 15% reaching a viral load of less than 1000 cp/ml. In addition, Afrashteh et al.^38^, also showed a significantly higher risk (aOR: 7.34 [95% CI 1.16, 46.21]) of late ART initiation among IDUs living in south of Iran. Moreover, Rodríguez-arenas et al.^51^, found that among IDUs ART-naive patients had a 33% lower probability of initiating ART compared to MSM (adjusted hazard ration (aHR): 0.67 [95% CI 0.57,0.79]) and heterosexual (aHR: 0.95 [95% CI 0.84, 1.09]) ART-naive patients. Several reasons have been described for such significant delay in IDUs, including disadvantaged social situation, economic difficulties, lack of family and social support, irregular adherence to treatment, improper social services, and occasional preference by professionals to delay ART until addiction control is ensured^51^. Regarding delayed ART initiation in HIV patients who had HIV-positive spouses, it has also been proposed that HIV-infected patients who were in long-term relationships with their HIV-positive partners were more likely to present late for HIV care, which might be due to a perception of lower risk^57^.

In the MSM group, a decline was observed in the mean duration to initiate ART, significantly at higher percentiles. The Collaboration of Observational HIV Epidemiological Research in Europe (COHERE) study of approximately 84,000 individuals living with HIV from 35 European countries also reported a decrease in late presentation over time, especially among MSM; however, it increased for male IDUs and female heterosexuals^58^. While there is an extreme fear of stigma and discrimination among MSM individuals in Iran, to mitigate the fear of the negative social consequences of HIV infection, establishment of anonymous voluntary counseling and testing (aVCT) for HIV might have a role in the observed decrease in our study^41^. This result might also reflect the greater perceived risk of and awareness about HIV/AIDS among MSM, leading to early diagnostic, preventive, and treatment services^26,59^.

## Conclusion

Our findings are consistent with previous works, indicating that male gender, diagnosis after the implementation of the 90-90-90 UNAIDS goals, being IDU, and lower education levels are associated with a longer duration to initiate ART. Differences in some determinants, such as younger age, might be attributed to socioeconomic and cultural variations affecting care-seeking behaviors in different regions. Despite improvements in duration to initiate ART after implementing the WHO’s 2016 program in Iran as well as freely available ART service, delayed ART initiation remains highly probable among vulnerable sociodemographic groups. Our national-level findings might suggest health policymakers establish additional care plans for these groups, including early HIV testing, early ART initiation, HIV preventive measures, active care surveillance, educational programs, cultural interventions, and appropriate incentives.

### Limitations and strong points

One of the study limitations was that it was only conducted on the patients with complete data on the duration of diagnosis and ART initiation. This study might be further limited by lack of availability of other potential independent variables. However, strengths include the use of national HIV/AIDS data with a large sample size and the application of the quantile regression model. While, the traditional regression model assumes the similar effects of independent variables on the distribution of the dependent variable, the varied coefficients in different distributions of ART initiation justified the use of quantile regression in this study.

### Supplementary Information

Supplementary Information.

## Data Availability

The datasets used and/or analyzed during the current study available from the corresponding author on reasonable request.
