# Supplementary material for: Factors associated with late initiation of antiretroviral therapy in Iran’s HIV/AIDS surveillance data
Source: Sci Rep. 2024 Jan 2;14:199. doi: 10.1038/s41598-023-50713-0 (PMC10761711; doi:10.1038/s41598-023-50713-0)
Supplement: Supplementary file 1 — Supplementary Information. [file 41598_2023_50713_MOESM1_ESM.docx]

**Supplementarty figure 1.** Visual inspecton (Q-Q plot) of the dependent variable distribution. Shapiro-Wilk W test for normality showed non-normal distribution of the dependent variable (Test results = W statistic: 0.72311, V statistic: 2170.315, Z statistic: 20.846, P <0.001)

| **Supplementary table 1.** Univariable linear regression for determining the association between the independent variables and duration (days) to initiate ART |
| --- |

| Variables | Group | Crude coefficient | SE | 95% CI | P |
| --- | --- | --- | --- | --- | --- |
| Gender ^†^ | Male | Reference | - | - | - |
|  | Female | -334.61 | 20.13 | -374.07, -295.15 | <0.001 |
| Education level ^†^ | Illiterate | Reference | - | - | - |
|  | Elementary | 138.16 | 29.17 | 88.98, 195.33 | <0.001 |
|  | Junior high school | 176.53 | 28.27 | 121.11, 231.94 | <0.001 |
|  | Senior high school | -10.2.68 | 30.15 | -161.77, -43.58 | 0.001 |
|  | Higher-than-diploma | -336.62 | 39.75 | -414.52, -258.71 | <0.001 |
| Years of report ^†^ | Before 2016 | Reference | - | - | - |
|  | After 2016 | -1026.19 | 19.38 | -1064.17, -988.21 | <0.001 |
| Age ^†^ | - | -21.78 | 0.85 | -23.45, -20.11 | <0.001 |
| Injection drug user ^†^ | No | Reference | - | - | - |
|  | Yes | 573.37 | 18.37 | 537.37, 609.37 | <0.001 |
| Men who have sex with men (MSM) ^†^ | No | Reference | - | - | - |
|  | Yes | -227.06 | 43.38 | -312.08, -142.04 | <0.001 |
| Heterosexual ^†^ | No | Reference | - | - | - |
|  | Yes | -66.72 | 19.74 | -105.43, -28.02 | 0.001 |
| Having an HIV-positive spouse ^†^ | No | Reference | - | - | - |
|  | Yes | -109.01 | 25.18 | -158.37, -59.66 | <0.001 |
| Having a spouse with high-risk behaviors ^†^ | No | Reference | - | - | - |
|  | Yes | -360.74 | 43.52 | -446.05, -275.43 | <0.001 |
| Other routes of transmission ^†^ | No | Reference | - | - | - |
|  | Yes | -124.49 | 24.28 | -172.08, -76.90 | <0.001 |

| ^†^ Included into the multivariable linear regression model. |
| --- |
